# Leisure activity associated with cognitive ability level, but not cognitive change

**DOI:** 10.3389/fpsyg.2014.01176

**Published:** 2014-10-14

**Authors:** Alan J. Gow, Kirsten Avlund, Erik L. Mortensen

**Affiliations:** ^1^Department of Psychology, School of Life Sciences, Heriot-Watt UniversityEdinburgh, UK; ^2^Section of Social Medicine, Department of Public Health, University of CopenhagenCopenhagen, Denmark; ^3^Center for Healthy Aging, University of CopenhagenCopenhagen, Denmark; ^4^Danish Aging Research Center, Universities of Southern DenmarkAarhus and Copenhagen, Odense, Denmark; ^5^Unit of Medical Psychology, Section of Environmental Health, Department of Public Health, University of CopenhagenCopenhagen, Denmark

**Keywords:** cognitive aging, leisure activity, preserved differentiation, differential preservation, causality

## Abstract

Although activity participation is promoted as cognitively protective, critical questions of causality remain. In a cohort followed every 5 years from age 75 to 85 years, potential reciprocal associations between level and change in leisure activity participation and level and change in cognitive abilities were examined. Participants in the Glostrup 1914 Cohort, a longitudinal study of aging, completed standardized cognitive ability tests and reported their leisure activity participation (11 activities defined a leisure activity score) at ages 75, 80, and 85. Higher leisure activity was associated with higher cognitive ability (significant correlations ranged from 0.15 to 0.31, *p* < 0.05). Between ages 75 and 85, participation in leisure activities and cognitive ability declined significantly. Growth curve models, which provided latent variables for level of and 10-year change in both leisure activity and cognitive ability, confirmed the positive association between levels of leisure activity and cognitive ability (path coefficient = 0.36, *p* < 0.001); however, neither leisure activity level nor change in leisure activity were associated with cognitive change. Although a positive association between leisure activity and cognitive ability was reported—the likely precedents of this are discussed—there was no evidence that a higher level or maintenance of leisure activity was protective against cognitive decline across a 10-year follow-up.

## INTRODUCTION

In searching for factors that might protect against age-related cognitive changes, perhaps one of the most scrutinized areas is leisure-time activity participation. Across numerous studies, associations between greater engagement in leisure activities and better cognitive outcomes have been reported (Hertzog et al., 2009). However, a critical issue remains concerning the nature of these associations: do they reflect a cognitively protective effect of activity participation, or are they an outcome of those of higher cognitive ability always being, and subsequently able to remain, more intellectually and socially active? To establish that activity participation is cognitively protective, the crucial association is, therefore, whether the level of participation in leisure activities, or change in this, is not only associated with the level of cognitive ability, but also reduces or delays cognitive decline. The potential reciprocal associations between leisure activity and cognitive aging were examined in a longitudinal cohort, assessed across the eighth and ninth decades.

Adults participating in more leisure-time activity generally have higher cognitive ability, an association replicated not only cross-sectionally but in longitudinal studies with follow-ups ranging from years to decades (Hultsch et al., 1993, 1999; Richards et al., 2003; Wilson et al., 2003a,b; Schaie, 2005). For current purposes, leisure activities will be broadly considered as those with combinations of intellectual and social demands, or a degree of physical exertion. The association between physical activity and cognitive aging, though equally well-replicated, is often considered separately (Hertzog et al., 2009); some explanatory mechanisms might be shared while others are unique to the physical aspect, including improved cardiovascular health, for example. The current approach was to consider a range of common activities across the different domains together, as this might more accurately reflect the ways in which individuals are engaged in hobbies and interests, rather than relatively arbitrary decisions about what might be a purely social activity, versus a purely intellectual or physical activity, for example. Most activities will be composed of a particular combination of intellectual, social and physical stimulation to create an overall engagement profile for the individual. It is this overall activity engagement which is the current focus. The ubiquity of the leisure activity-cognitive ability association has been interpreted as suggesting that increasing or maintaining engagement in leisure activities in old age can be considered one pathway to reduce or delay cognitive aging (Hertzog et al., 2009).

Indeed, this has underpinned the development and testing of lifestyle-based interventions in which participants are randomized to different activity groups (versus no contact or active control comparisons), to examine the effect of increasing engagement on subsequent cognitive change. The work of Park et al. (2014) is at the leading edge of this, highlighting potentially beneficial effects of undertaking novel activities such as digital photography or quilting over about 3 months, for example. Beneficial effects of participating in such productive activities have been reported for episodic memory function, versus increased social interaction with no specific cognitive novelty (Park et al., 2014). While the intervention literature is important, particularly as it seeks to translate observed activity-cognition associations into real-world benefits, it will not be discussed in detail given that the current study is purely observational. Hertzog et al. (2009) provide a detailed critique of both aspects of this literature.

Assuming for present purposes that it is leisure activity which affects later cognitive change, within the cognitive aging literature the reported benefit is often explained in terms of the cognitive stimulation from increased activity participation providing a “mental workout” for the brain. That is, participation in such activities affords a more complex environment (Schooler, 1984), which engenders the development, enhancement, and/or maintenance of cognitive skills through their continued deployment. This is commonly cited within the (dis)engagement, or more colloquially the “use it or lose it,” theory of cognitive aging (Salthouse, 2006). The literature in this area is substantial, and given global aging trends, ever-increasing. Hertzog et al. (2009) provided a thorough overview, with a detailed consideration of the evidence for how lifestyle factors might influence cognitive aging.

In all studies examining associations between leisure activity and cognitive aging, there are a number of important methodological and conceptual considerations (Hertzog et al., 1999; Pushkar et al., 1999; Salthouse, 2006; Bielak, 2010; Gow et al., 2012a). Some relate to differences between associations of leisure activities at a general versus a more domain-specific level (different domains being intellectual, social, or physical, for example) while another issue is whether these associations might be with general versus specific cognitive abilities. These are partly determined by the measures included (that do or do not afford the definition of general versus specific leisure/cognitive factors) or the researchers’ aim to focus on general versus specific associations. Though important, this and similar methodological issues (length of follow-up, for example) can be addressed in many of the large, well-phenotyped cohorts examining cognitive aging. Bielak (2010) discussed these, and further highlighted perhaps the most pressing issue: determining causality. Given an association, even when that might be longitudinal, is leisure activity the preceding factor? Debate over the extent to which studies have adequately addressed this issue is the main reason why the “use it or lose it” hypothesis, with respect to leisure activity participation and cognitive aging, cannot be universally accepted (Salthouse, 2006; Bielak, 2010).

The causality issue is central to understanding how leisure activities and cognitive abilities might develop and change in tandem. Indeed, analysis and reinterpretation of reported associations from large-scale studies have focussed specifically on the issue (Hertzog et al., 1999; Hultsch et al., 1999; Pushkar et al., 1999). The ongoing debate is often phrased within the context of differential preservation versus preserved differentiation (Salthouse, 2006; Bielak, 2010; Bielak et al., 2012). In searching for determinants of cognitive aging, researchers aim to identify evidence of differential preservation: dependent on the level of a given factor (such as leisure activity), an individual’s cognitive abilities are protected to a greater or lesser degree across time. However, researchers must acknowledge the possibility of preserved differentiation, often also referred to as reverse causation; contemporaneous associations might be driven by a shared antecedent variable. In terms of cognitive aging, prior cognitive ability is a major confounder, given the high stability of cognitive ability across the lifecourse (Deary et al., 2000), and that it is a determinant of lifestyle choices including leisure activity (Gow et al., 2012b).

Studies addressing the likelihood of reverse causation have, where possible, accounted for an early measure of prior ability (Gow et al., 2012b; Richards et al., 2003). For example, leisure activity remained associated with the level of cognitive ability in midlife after adjusting for a measure of childhood cognitive ability (Richards et al., 2003), although in another study, the association between leisure activity and cognitive ability (general ability, processing speed, and memory) at age 70 was eliminated (Gow et al., 2012b). Few aging studies have measures of cognitive ability from childhood or early adulthood, though alternative approaches have provided fundamental insights.

Most studies examine leisure activity and cognitive ability at a given age, and then assess only the change in cognitive ability from that point onward. However, if leisure activity is also assessed across time, it is possible to examine potential reciprocal associations. In a coordinated analysis across four large studies, although there were the expected cross-sectional associations between leisure activity and cognitive ability (reasoning, fluency, memory, and semantic knowledge), baseline leisure activity was not associated with change in cognitive ability, with follow-ups ranging from 8 to 21 years (Mitchell et al., 2012). There was some evidence for an association between change in leisure activity and change in cognitive ability, but the specific analyses did not allow causality to be unraveled. It is specifically the cross-lagged effects which are the key to the question of how activity and cognitive abilities might affect one another across old age. That is, does activity level affect subsequent cognitive change, or does cognitive ability level predict the likely maintenance or withdrawal from activities? For example, Bielak et al. (2012) reported that although individuals with higher leisure activity participation had higher cognitive ability, there was no association with cognitive change over 8 years (Bielak et al., 2012).

The dearth of studies addressing this issue is a result of leisure activity often only being considered at a baseline assessment, while cognitive ability may be assessed on multiple occasions across longitudinal follow-ups. The current study addressed this in the Glostrup 1914 Cohort. Previous analyses suggested that leisure activities at ages 50, 60, and 70 were associated with the level of cognitive ability from ages 60 to 80 (Gow et al., 2012c). However, the associations were attenuated after controlling for cognitive ability at age 50, implicating preserved differentiation in the associations. There were no associations between level of leisure activity participation and cognitive change between ages 60 and 80. Differences in the assessment of leisure activity over the decades meant it was not possible to consider more nuanced reciprocal associations between level and change in both leisure activity and cognitive ability. More recent waves retained a consistent assessment of leisure activity, therefore the current analysis specifically examined reciprocal associations between leisure activity and cognitive aging at ages 75, 80, and 85.

## MATERIALS AND METHODS

### PARTICIPANTS

All participants were born in 1914 and were members of the Glostrup 1914 Cohort (Osler et al., 2010). Participants were initially recruited at age 50 (*N* = 802) from Glostrup, a suburb of Copenhagen; the sample was randomly selected and was representative (sex and social class) of the Danish population (Schroll, 1982, 2003). Participants were subsequently assessed every 10 years to age 70, and every 5 years thereafter to age 95, with new participants recruited at ages 70 and 75 (Osler et al., 2010). The current analyses used data from the 75, 80, and 85 year assessments as they combined consistent assessments of both cognitive ability and leisure activities over time, with sample sizes of 576 at 75 (Avlund et al., 1995), 505 at 80, and 243 at 85. The waves reported were conducted under relevant ethical committee approval from the Scientific Ethics Committee for the Capital Region of Denmark; participants provided written, informed consent and data collection was in accordance with the World Medical Association Declaration of Helsinki.

### COGNITIVE ABILITY

At each assessment, participants generally completed 11 subtests from the Danish translation of the WAIS (Wechsler, 1955): information, comprehension, arithmetic, similarities, digit span, vocabulary, digit symbol, picture completion, block design, picture arrangement, and object assembly (Mortensen and Kleven, 1993; Mortensen and Høgh, 2001; Krabbe et al., 2009). However, at the age 75 assessment, only a sub-sample completed the psychological measures which for cognitive ability consisted of digit span and digit symbol (Mortensen and Høgh, 2001). The current analyses therefore used these two tests to define cognitive ability at ages 75, 80, and 85, which were scaled to age 50 norms to control for age at time of testing.

### ACTIVITY PARTICIPATION

At ages 75, 80, and 85, participants were asked about their participation in 18 leisure activities, such as “participation in family occasions,” “activities of clubs or associations,” and “playing cards.” A further item (“go to watch sport games”) was only included in the age 75 and 80 assessments, so was not considered further. Participants reported their frequency of participation in each activity using a 6-point scale: never, rarely, a few times a year, monthly weekly, or daily.

### COVARIATES

Basic covariates known to be associated with cognitive ability or aging and included in previous analyses were considered: sex, education, and social class (Gow et al., 2012c). Education was the combined score of participant’s school education (on a 3-point scale, from primary to upper secondary) and vocational training (on a 5-point scale, from no vocational training to academic; Mortensen and Høgh, 2001). Participants were assigned a social class category using a six category system according to their ooccupational information (Svalastoga, 1959).

### STATISTICAL ANALYSES

The 18 leisure activities assessed at ages 75, 80, and 85 were analyzed by principal components analysis (PCA) to investigate the underlying structure and generate summary scores, using IBM SPSS Statistics Version 20.0 (IBM, Somers, NY, USA). Growth curve models were used to examine reciprocal associations between level and change in leisure activity and cognitive ability, using Mplus Version 7 (Muthen & Muthen, Los Angeles, CA, USA). Prior to the analysis, continuous variables were standardized. In some of the models, the covariance coverage was reduced from the default of 10% but not below the suggested minimum threshold of 5%.

For cognitive ability, a latent general cognitive ability factor was defined at ages 75, 80, and 85 by the two cognitive tests: digit symbol and digit span. Before completing the main analyses, it was necessary to check for measurement invariance across ages 75–85 in the general cognitive ability factor, using a four-stage process as described previously (Gow et al., 2012c). In summary, a model was run with all parameters free to vary, followed by models with factors loadings, then residual variance and finally intercepts constrained equal. Deterioration in model fit from the least to most constrained models would suggest a lack of measurement invariance (Meredith, 1993). Model fit indices reported are the root mean square error of approximation (RMSEA) and the comparative fit index (CFI).

In growth curve models, latent terms are generated for intercept (level) and slope (change over time). There were therefore terms for intercept and slope for both leisure activity and cognitive ability. Associations between the level and change in both leisure activity and cognitive ability were examined. Participants were included if they contributed any data at the age 75, 80, and 85 assessments using full information maximum likelihood (FIML; Arbuckle, 1996).

## RESULTS

### SAMPLE DESCRIPTIVES

**Table 1** displays descriptive data for the Glostrup 1914 Cohort at ages 75, 80, and 85, for participants with at least one of the cognitive tests at any given age. More detailed comparisons across waves for leisure activity/cognitive ability are described below.

**Table 1:** The Glostrup 1914 Cohort: sample descriptives at ages 75, 80, and 85.

	Age 75 (*N* = 401)	Age 80 (*N* = 344)	Age 85 (*N* = 156)
Sex (% male)	48.4	48.0	40.4
Education	3.5 (1.4)	3.5 (1.4)	3.6 (1.4)
Social class	6.5 (1.0)	6.3 (1.0)	6.3 (1.0)
Leisure activity	29.6 (7.5)	30.2 (7.8)	26.0 (8.0)
Digit span	9.8 (1.8)	9.1 (1.4)	8.8 (1.5)
Digit symbol	33.9 (1.3)	31.2 (1.3)	25.0 (1.2)

*For descriptive purposes, sample size was determined by the presence of at least one of the cognitive variables at any given age. Education was an overall score combining school education on a 3-point scale (primary to upper secondary) and vocational training on a 5-point scale (no vocational training to academic). Higher scores represent more education/training. Social class was coded according to Svalastoga (1959), producing six categories labeled strata three (most professional) to eight (unskilled occupations). Leisure activity was the sum of the 11 activity items described in the principal components analysis (PCA). For leisure activity and cognitive ability, the data in the table represent data from participants with complete data across all three waves for illustrative purposes, though the modeling approach included participants with data at any wave.*

### PCA OF LEISURE ACTIVITY ITEMS

The 18 leisure activity items completed at ages 75, 80, and 85 were analyzed by PCA. In the analysis at age 85, the item “outdoor hobbies” had an individual measure of sampling adequacy (MSA) of 0.42 (Kaiser, 1974); the item was therefore removed across all ages, and the PCA were repeated on the remaining 17 items. At each age, a clear first unrotated component was suggested, explaining 17.5%, 19.5%, and 18.9% of the variance, respectively. All items loaded positively on this first unrotated component (**Table 2**), except “repairs in home/car” at age 85 which had a loading of 0.00. Eleven items that loaded over 0.30 across all three ages were summed to define leisure activity scores at ages 75, 80, and 85, with internal reliabilities (Cronbach’s α) of 0.65, 0.68, and 0.66 respectively.

**Table 2:** Principal components analysis of leisure activity items.

	Age 75 (*N* = 736)	Age 80 (*N* = 489)	Age 85 (*N* = 215)
Theatre, movies, concerts, art exhibitions	**0.58**	**0.61**	**0.64**
Travel in foreign countries	**0.57**	**0.56**	**0.50**
Travel in home country	**0.53**	**0.61**	**0.56**
Active participation in sports	**0.50**	**0.43**	**0.36**
Adult education with academic subjects	**0.50**	**0.61**	**0.56**
Activities of clubs or associations	**0.46**	**0.48**	**0.52**
Participation in family occasions	**0.45**	**0.52**	**0.40**
Use of library	**0.44**	**0.48**	**0.43**
Participation in church activities	**0.43**	**0.40**	**0.61**
Taking walks	**0.42**	**0.43**	**0.45**
Reading	**0.41**	**0.48**	**0.49**
Gardening	**0.37**	0.29	0.17
Adult education about manual subjects	**0.30**	0.16	0.27
Playing cards	0.26	**0.32**	0.29
Repairs in home and car	0.24	0.14	0.00
Needlework	0.21	**0.34**	**0.36**
Bingo	0.13	0.21	0.25

*The item “outdoor hobbies” was excluded due to a low measure of sampling adequacy (MSA) in the age 85 PCA. Item loadings on the first unrotated component are shown at each age; loadings over 0.30 are highlighted in bold. The 11 items which loaded over 0.30 at all three ages were summed to define leisure activity scores for ages 75, 80, and 85.*

In the PCA, the scree plots and Eigenvalues greater than 1 criterion further suggested that up to six factors might be extracted, with three being most consistent across waves. Three factor solutions were extracted with varimax rotation. Although the first rotated factors were relatively consistent across ages (though defined by fewer items and therefore accounting for less variance than the first unrotated components), the second and third rotated factors differed across ages, rendering cross-wave comparisons impossible. Only the leisure activity scores defined by the 11 items loading consistently on the first unrotated components were therefore considered further.

### LEISURE ACTIVITY DESCRIPTIVES

Leisure activity scores were significantly and positively intercorrelated, ranging from 0.50 (*p* < 0.001) between ages 80 and 85, to 0.62 (*p* < 0.001) between ages 75 and 80 (**Table 3**); being more active at any age at was associated with higher activity at the others also.

**Table 3:** Associations between leisure activity and cognitive ability from 75 to 85 years.

	Leisure activity 75	Leisure activity 80	Leisure activity 85	Digit symbol 75	Digit span 75	Digit symbol 80	Digit span 80	Digit symbol 85
Leisure activity 80	0.62***							
Leisure activity 85	0.51***	0.50***						
Digit symbol 75	0.28***	0.30***	0.19					
Digit span 75	0.16***	0.23***	0.23*	0.45***				
Digit symbol 80	0.15*	0.28***	0.31***	0.80***	0.43***			
Digit span 80	0.23***	0.21***	0.24**	0.33***	0.60***	0.43***		
Digit symbol 85	0.17*	0.06	0.18*	0.35***	0.53***	0.48***	0.54***	
Digit span 85	0.22*	0.19*	0.19*	0.79***	0.43***	0.80***	0.42***	0.48***

N = 71–422. Leisure activity 75, 80, and 85 were computed by summing the 11 items which consistently loaded over 0.30 on the first unrotated component the PCA across all three ages (***Table 2***). *p < 0.05, **p < 0.01, ***p < 0.001.

The mean leisure activity scores for ages 75, 80, and 85 were, respectively: 26.5 (SD = 8.0), 26.9 (8.1), and 25.7 (8.1). These data describe all available participants at any given wave (*N* = 736, 489, and 215). When only participants with leisure activity data at all three waves were considered (*N* = 175), the means were: 29.6 (7.5), 30.2 (7.8), and 26.0 (8.0), as shown in **Table 1**. A repeated-measures ANOVA suggested leisure activity scores were significantly different across time [*F*(1.86,323.20) = 33.45, *p* < 0.001]; *post hoc* comparisons identified a significant reduction in leisure activity scores between ages 75 and 85, and ages 80 and 85 (*p* < 0.001).

Associations between leisure activity and cognitive ability scores are reported in **Table 3**. All correlations were positive but small, with the significant associations ranging from 0.15 to 0.31 (*p* < 0.05); in general, individuals participating in more leisure activities at any given age tended to have higher cognitive ability.

### COGNITIVE ABILITY DESCRIPTIVES

The mean cognitive ability scores are also shown in **Table 1**. Again, these are for participants with full data across all three waves for illustrative purposes (though the modeling included participants with data at any wave). Performance on both cognitive tests declined across time [F(2,148) = 16.34, *p* < 0.001, for digit span and F(2,120) = 79.14, *p* < 0.001, for digit symbol]. *Post hoc* tests revealed that digit span decreased significantly between ages 75 and 80, and ages 75 and 85 (*p* < 0.001), and digit symbol decreased at each occasion of assessment (*p* < 0.001).

### MODELING LEVEL AND CHANGE IN LEISURE ACTIVITY AND COGNITIVE ABILITY

The main growth curve models described below include both leisure activity and cognitive ability; however, models were first produced separately for cognitive ability and leisure activity to examine general change and one- versus two-slope solutions. The cognitive ability model is described first. As a general cognitive ability factor was to be defined by two cognitive tests across three waves of assessment in the growth curve model, measurement invariance was assessed. In the first model with all parameters varying freely, the model was not identified; moving through the more constrained models (factors loadings, then residual variance and finally intercepts held equal), resulted in improved model parameters CFI = 0.91, AIC = 4432.11, and RMSEA = 0.084 (90% C.I. = 0.062–0.108) therefore suggesting measurement invariance, though the parameters would usually be considered as indicating suboptimal model fit.

**FIGURE 1:**
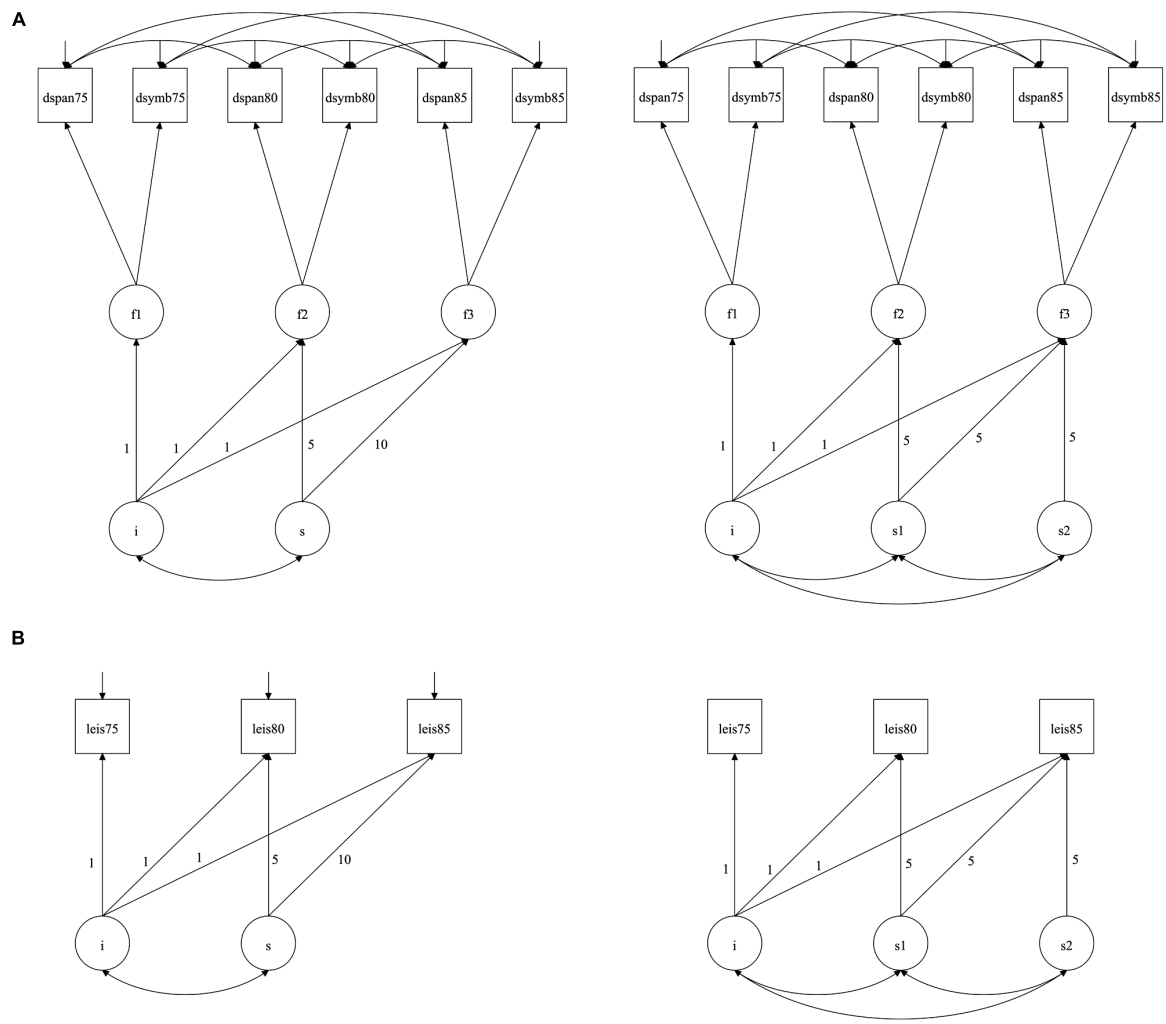
**(A)** Model of cognitive change from age 75 to 85: one-slope (left) and two-slope solutions; **(B)** Model of leisure activity change from age 75 to 85: one-slope (left) and two-slope solutions. Note. The diagrams are purely for illustrative purposes, with significant path coefficients described in the Results. In the models (adapted from Gow et al., 2012c), measured variables are represented by rectangles, and latent variables by circles. The principal outcome variables in the model are intercept (the level of leisure activity/general cognitive ability) and slope (the change in leisure activity/general cognitive ability across time). Measured variables have fixed contributions to the intercept. In the one-slope solutions (left), change across time is considered as a single parameter, with 5-year change to age 80 and a further 5-year change to age 85 (the fixed contributions to slope; 5 and 10) represent the number of years since the initial testing occasion, age 75 in this model. In the two slope solution, two distinct periods of change are considered: firstly from age 75 to 80, and then from age 80 to 85. *i* = intercept and *s* = slope; f1, f2, and f3 represent latent general cognitive ability defined by the two cognitive tests at ages 75, 80, and 85 respectively; leis75, leis80, and leis85 represents the leisure activity scores at ages 75, 80, and 85 respectively.

When the intercept and slope parameters were added to the cognitive ability model (illustrated in **Figure 1A**), the fit parameters improved. Both one- and two-slope solutions were examined, and suggested significant cognitive decline over time: in the one-slope solution, the decline was -0.025 (*p* < 0.001) from age 75 to 85, though there was no significant variance in this; for the two-slope solution, there was decline from age 75 to 80 (-0.024, *p* = 0.001) and age 80 to 85 (-0.027, *p* = 0.004), although again the variance in slope was not significant. For both one- and two-slope solutions, there was significant variance in the intercept (0.231, *p* < 0.001). There were no associations between intercept and slope(s) in either of the models suggesting that the level of cognitive ability was not associated with cognitive change.

In the one-slope leisure activity model (**Figure 1B**, left panel), there was a mean decline of -0.027 (*p* < 0.001) from age 75 to 85, though the variance in this was not significant. There was, however, significant variance in the level of leisure activity (0.822, *p* < 0.001). When a two-slope solution was examined (**Figure 1B**, right panel), there was decline from age 75 to 80 (-0.015, *p* = 0.045), and from age 80 to 85 (-0.055, *p* < 0.001), with significant variance of 0.029 and 0.041 (*p* < 0.001), respectively.

**FIGURE 2:**
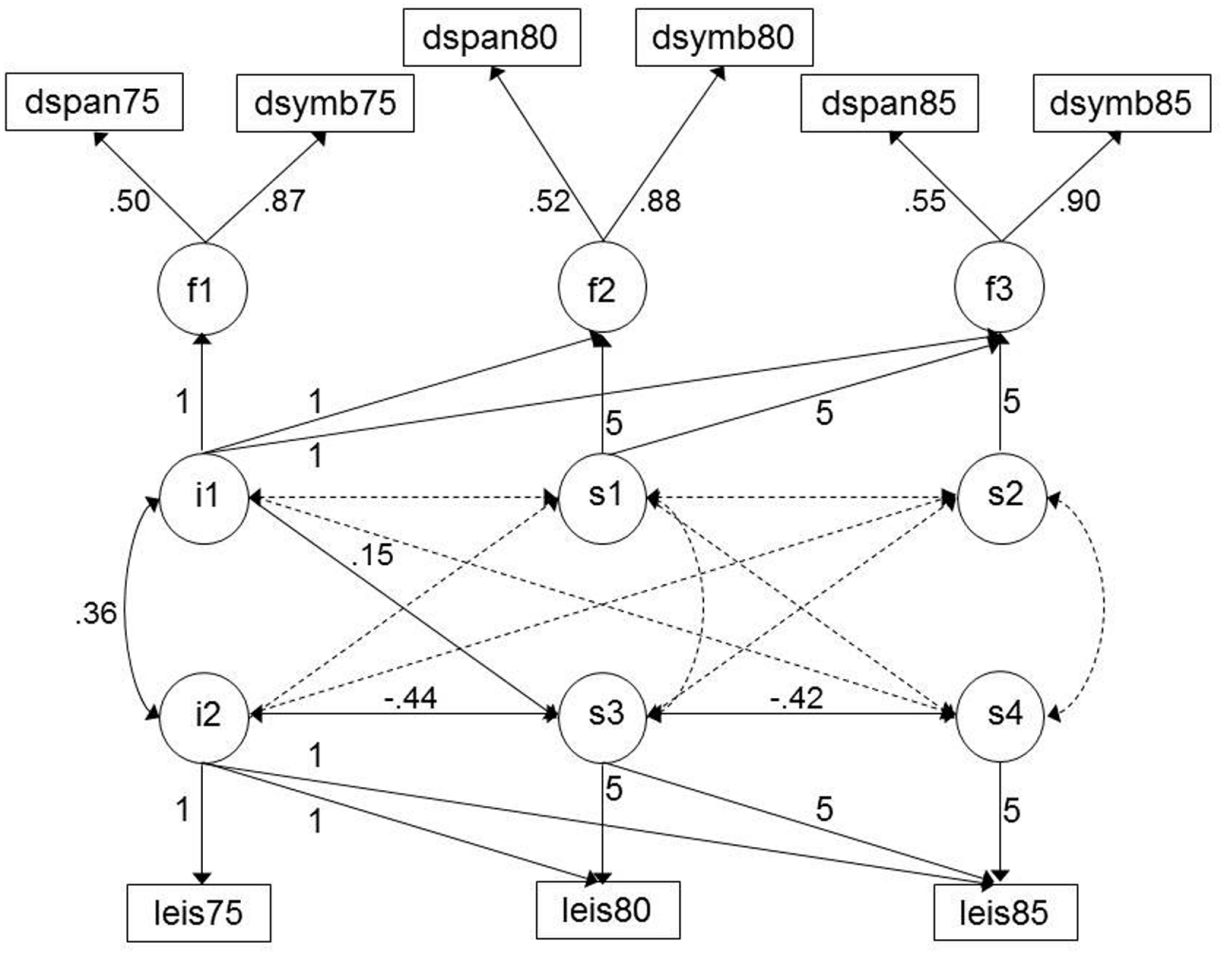
**Model of cognitive and leisure activity change from age 75 to 85: two-slope solution.** See note **Figure 1**. Significant paths are highlighted by solid lines. Correlations between leisure activity/cognitive tests are omitted for clarity but were included in the model. Figures with decimal points are the standardized estimates generated by the model.

In combining the cognitive and leisure activity models, the two-slope model was calculated as it provided the best description of change in leisure activity over time (two significant slopes, each with significant variance). The model fit for the combined two-slope leisure activity-cognitive ability model was CFI = 0.999, RMSEA = 0.008, 90% C.I. = 0.000–0.033. **Figure 2** represents this model, with the significant path coefficients listed. The intercepts of leisure activity and cognitive ability were associated (0.36), replicating the correlations described above. There were no associations between leisure activity (level or change) and change in cognitive ability. However, level of cognitive ability was associated with change in leisure activity from ages 75 to 80 (0.15): individuals with a higher level of cognitive ability showed less decline in their leisure activity participation.

In a final model, the covariates sex, education, and social class were added; this resulted in a slight reduction in model fit due to missing data on, particularly, social class (CFI = 0.969, RMSEA = 0.041, 90% C.I. = 0.015–0.063). The intercepts of leisure activity and cognitive ability remained associated (0.27), however, the association between level of cognitive ability and change in leisure activity was no longer significant. In terms of the covariates, education was positively associated with the levels of cognitive ability and leisure activity (0.37 and 0.14, respectively), and social class was negatively associated (-0.44 and -0.18, respectively; higher levels of activity being associated with more professional occupations). Sex was associated with leisure activity intercept (0.28) whereby women had a higher level. None of the covariates were associated with change in either leisure activity or cognitive ability.

## DISCUSSION

With three waves of data collected across a 10-year period, associations between level and change in both leisure activity and cognitive ability were examined in the Glostrup 1914 Cohort. Although associations between the levels of leisure activity and cognitive ability were reported—individuals participating in more activity had higher cognitive ability or vice versa—there was no evidence of an association between level of leisure activity (or change in this) and subsequent cognitive change. The current study does not support leisure activity as a cognitively protective factor (other than by being associated with level earlier in the lifecourse discussed below) across the eighth and ninth decades.

Taking the general association between leisure activity and cognitive ability first, the current findings replicate those widely reported in the literature (Hertzog et al., 2009). Individuals participating in more leisure activities generally score higher on tests of cognitive ability. In the current study, this held when covariates including sex, education, and social class were considered. However, in studies which have been able to account for an earlier measure of cognitive ability, this association was eliminated suggesting that it may be an outcome of preserved differentiation (Gow et al., 2012b,c). Another study also accounting for prior ability suggested that the association between leisure activity and cognitive ability remained, although it assessed adults in midlife (Richards et al., 2003). These findings are relevant to the nature of the contemporaneous association between leisure activity and cognitive ability. If assessed in old age, the associations may reflect only that individuals who were always more able remain so, and additionally are more active (preserved differentiation), or that it is earlier in the lifespan that activity has a cognitively enhancing effect (Richards et al., 2003). Though the current study cannot explore the likelihood of either explanation further, it is important to highlight the issue so that those with datasets covering early adulthood to midlife, and midlife to old age might more fully elucidate the underlying nature of the well-cited association (Salthouse, 2006). Leisure activity might still be considered cognitively protective, for example, if it increases the level of cognitive ability across the lifecourse, allowing an individual to enter later life at a higher level (Gow et al., 2012a).

The principal aim of the study, however, was to explore level and change in leisure activity and cognitive ability simultaneously. In this we replicated Bielak et al. (2012), for example, whereby leisure activity (level or change) was not associated with subsequent change in cognitive ability. If anything, it appeared to be cognitive ability being associated with change in leisure activity in the current study (although this was not significant after accounting for demographic covariates). It is these cross-lagged associations which are particularly relevant when examining change over time in two (or more) variables. Associations with level do not necessarily translate into associations with change; this is important for the desire to develop interventions based on observational work (Gow et al., 2012a).

### STRENGTHS AND LIMITATIONS

The current study benefitted from a consistent assessment of both cognitive ability and leisure activity across three waves of assessment. Though the cohort had been followed-up prior to the waves analyzed here, alterations in activity assessment over time meant that it was possible to examine change in this with cognitive change (Gow et al., 2012c). The use of the later, more standardized assessments is balanced by the smaller sample size, which reduced further due to attrition across the follow-up. The analytical procedure, however, allowed all participates to provide data, even where they were missing on more than one occasion.

Attrition and practice/retest effects were not specifically modeled, though there are complex issues in attempting to extract these effects from aging, for example (Hoffman et al., 2011). The Glostup 1914 Cohort is, however, a year of birth cohort, which minimizes the confounding of chronological age which would usually be present in large cohort studies. Given the assessments included, the current study only considered an overall marker of leisure activity and general cognitive ability. Other studies have considered distinct domains of cognitive function (Mitchell et al., 2012), or have also included specific factors for social, intellectual or physical activity, for example. Taking the cognitive ability assessment first, while combined in such a way as to extract the common variance, the two cognitive ability tests available do not reflect all domains of cognitive function. They are indicators of short-term memory or processing speed, and so the lack of an association between leisure activity and cognitive aging may be a consequence of how cognitive ability has been defined, rather than there being no association *per se*. The use of only two measures is likely to have affected the reliability of the modeling approach also. Studies with fuller batteries of cognitive function will be able to address these concerns, as well as the general or domain-specific nature of any protective effects of leisure activity participation, though the current results are broadly consistent with others following related analytical approaches (Bielak et al., 2012; Mitchell et al., 2012).

The current focus on a single leisure activity factor was data-driven, as it was the only factor which was consistently reproduced across the three waves. Given that other factor solutions were possible (albeit inconsistently so across waves), future work will focus on deconstructing this overall activity factor to examine the relative contributions of different domains of activity. That approach is, however, likely to be limited by the lack of measurement invariance in activity participation with increasing age suggested by the current analysis. The measure of leisure activity was based on frequency of participation, but there was no information about intensity, or the specific type of activity (reading a book and a magazine might not provide the same degree of cognitive stimulation, for example).

## CONCLUSION

That there exists an association between leisure activity and cognitive ability in old age is not in doubt (Hertzog et al., 2009), however, the true nature of this association remains in question. The current study cannot support leisure activity in old age as a cognitively enhancing factor. It is imperative to more fully understand the association: if differential preservation does occur, but simply much earlier in the lifecourse, our ideas of when to intervene to prevent age-related cognitive decline might need careful consideration; or if leisure activities are not cognitively protective and the association is purely the outcome of preserved differentiation, then efforts must be directed toward identifying more compelling intervention targets.

## AUTHOR CONTRIBUTIONS

Alan J. Gow conceived, conducted, and interpreted the analyses, and drafted and revised the manuscript. Kirsten Avlund and Erik L. Mortensen conceived and designed the acquisition of data, and revised the manuscript. All authors approved the final version for submission and are accountable for all aspects of the work.

## Conflict of Interest Statement

The authors declare that the research was conducted in the absence of any commercial or financial relationships that could be construed as a potential conflict of interest.
